# Prohibitin-mediated mitochondrial ubiquitination during spermiogenesis in Chinese mitten crab Eriocheir sinensis

**DOI:** 10.18632/oncotarget.21961

**Published:** 2017-10-23

**Authors:** Cong-Cong Hou, Chao-Guang Wei, Cheng-Peng Lu, Xin-Ming Gao, Wan-Xi Yang, Jun-Quan Zhu

**Affiliations:** ^1^ Key Laboratory of Applied Marine Biotechnology of Ministry of Education, School of Marine Sciences, Ningbo University, Ningbo 315211, China; ^2^ The Sperm Laboratory, College of Life Sciences, Zhejiang University, Hangzhou 310058, China

**Keywords:** prohibitin, mitochondria, ubiquitination, Eriocheir sinensis, crustacean

## Abstract

The sperm of *Eriocheir sinensis* has a cup-shaped nucleus that contains several mitochondria embedded at the opening of the cup. The acrosome vesicle also contains derivants of mitochondria. The mitochondria distribution pattern involves a decrease in the number and changes in the structure and transportation of these organelles. The decreased number of sperm mitochondria is achieved through autophagy or the ubiquitination pathway. Prohibitin (PHB), the mitochondria inner membrane protein, is an evolutionarily highly conserved protein, is closely associated with spermatogenesis and sperm quality control and is also a potential substrate of ubiquitination. However, whether PHB protein mediates the ubiquitination pathway of sperm mitochondria in crustacean animals remains poorly understood. In the present study, we revealed that PHB, a substrate of ubiquitin, participates in the ubiquitination and degradation of mitochondria during spermiogenesis in *E. sinensis*. To confirm this finding, we used shRNA interference to reduce PHB expression and an overexpression technique to increase PHB expression *in vitro*. The interference experiment showed that the reduced PHB expression directly affected the polyubiquitination level and mitochondria status, whereas PHB overexpression markedly increased the polyubiquitination level. *In vitro* experiments also showed that PHB and its ubiquitination decide the fate of mitochondria.

## INTRODUCTION

Spermiogenesis is the last stage of spermatogenesis, which includes acrosome formation, nuclear shaping and flagellation in mammals. For crustacean sperm, which have no flagellum, spermiogenesis only involves acrosome formation and nuclear shaping [1–3]. The mitochondrion, as a source of energy for sperm motion, displays different distribution patterns in different animal groups. Mitochondria were arranged in the midpiece, which is the formation of a regular mitochondrial sheath, or mitochondria were arranged in a plane in flagellated sperm [4]. Whereas in non-flagellated sperm, including the vast majority of crustaceans, particularly shrimp and crabs, mitochondria are embedded in a nuclear cup, or as a derivative integrated into the lamellar structure of the acrosome vesicle; or together with lysosomes and Golgi bodies to form lamellar complexes (LCx) involved in acrosome formation [1–3].

There are significant differences in the number of mitochondria and morphological changes in spermatogenic cells at different stages, as the number of mitochondria in early spermatid was higher, while the number of mitochondria in the late sperm began to decrease [5]. In spermatogenesis, sperm cells need to discard the vast majority of organelles and cytoplasm, including certain mitochondria, to form concentrated spermatid [4]. In mammals, mitochondria regulate mitochondrial degradation through specific autophagy-related pathways that are critical to maintaining cell homeostasis [6–8]. Sutovsky et al. showed that the ubiquitin protease system is associated with the selective degradation of sperm mitochondria [9, 10]. In recent years, studies have shown that mitochondria are ubiquitinated during mammalian spermiogenesis, and ubiquitin mitochondria are degraded by mitochondrial membrane protein PHB as a substrate [11].

PHB is a mitochondrial inner membrane protein that is highly conserved in evolution. PHB's general functions are related to cell cycle, cell signal transduction, apoptosis, and mitochondrial morphology and stability [12–14]. In mammals, PHB proteins contain two functionally related subunits, PHB1 and PHB2 [15]. PHB complexes are produced through interactions in the N-terminal hydrophobic region on the mitochondrial inner membrane [16, 17]. In mitochondria, PHB was also associated with mitophagy, stabilizing mitochondria genome through TFAM (Transcription Factor A, Mitochondrial), ROS (Reactive Oxygen Free Radical) reduction, mitochondrial morphology and mitochondrial apoptosis [18–22]. The function of PHB during spermatogenesis has been reported in many species, including *Rattus norvegicus* [23], *Saccharomyces cerevisiae* [24], *Caenorhabditis elegans* [25], *Procambarus clarkii* [26], *Cynops orientalis* [27], and *Boleophthalmus pectinirostris* [28]. In *C. orientalis* and *B. pectinirostris*, the distribution of *phb* gene or PHB protein in spermatogenesis was reported [25, 28]. In both *R. norvegicus* and *P. clarkia*, the PHB signal was detected in spermatogonia, spermatocytes and spermatids [23, 27]. In rat, the amount of PHB in leptotene spermatocytes was higher than in preleptotene spermatocytes [23]. PHB primarily functioned in nuclear shaping and acrosome formation in the spermiogenesis of *P. clarkia*, and the PHB signal was higher in spermatogonia than in secondary spermatocytes and mature sperm [27]. Additionally, PHB might play a more profound role in mediating mitochondrial ubiquitination [27]. However, whether PHB, as the ubiquitin substrate, participates in the mitochondria ubiquitination and degradation remains unclear.

**Figure 1:**
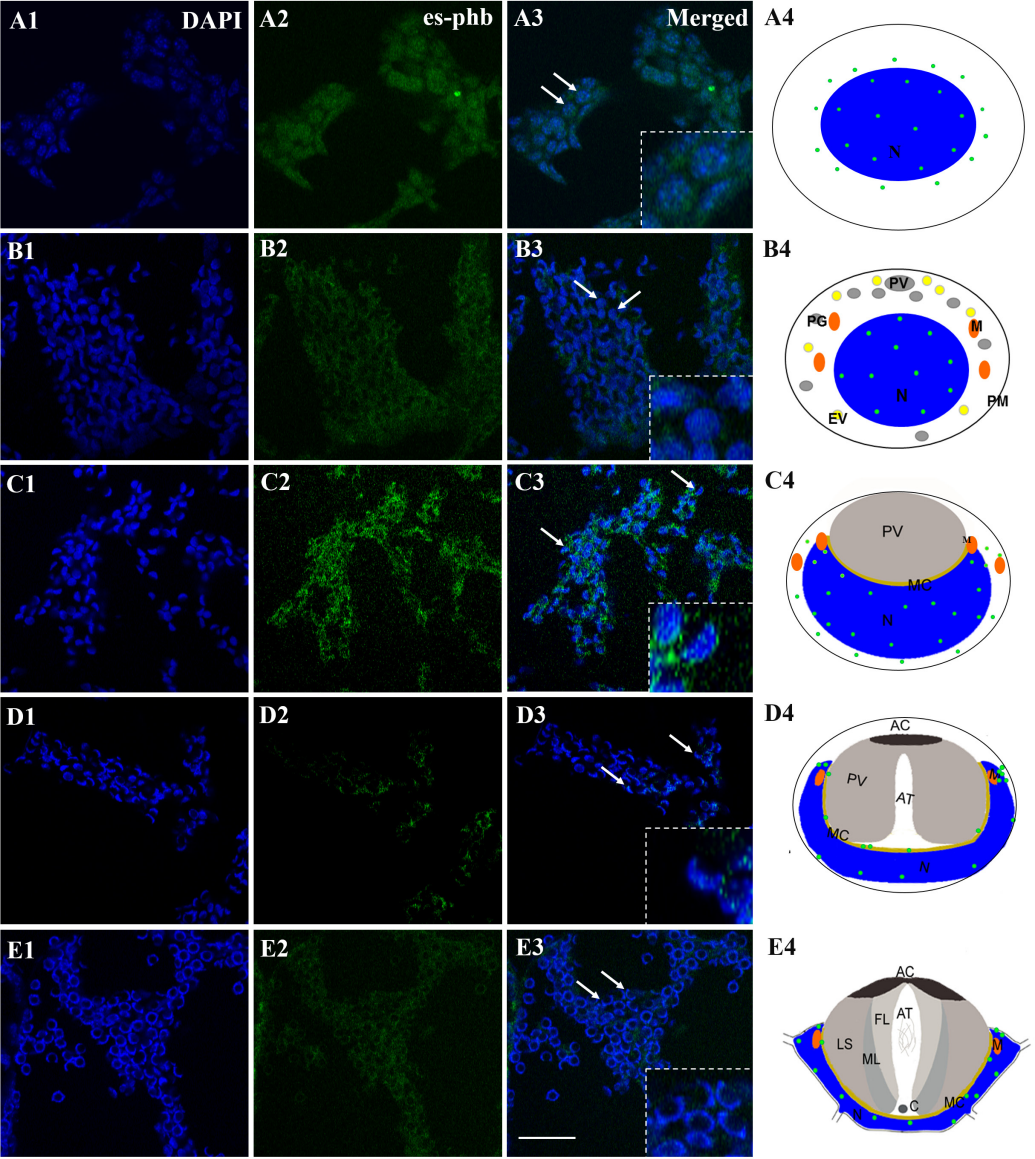
FISH of *phb* gene expression during the spermiogenesis of the Chinese mitten crab *E. sinensis* The *phb* mRNA primarily located in or around nucleus (arrows). In the round or elliptical spermatocytes, the *phb* mRNA signal in both the nucleus and cytoplasm (A3-A4, arrows). In round or elliptical spermatids, the *phb* mRNA signal was weakly distributed in the nucleus (B3-B4, arrows). In middle-stage spermatids, the nucleus becomes cup-like. The *phb* mRNA signals were distributed in the cup-like nucleus and gathered to one side in the cytoplasm. Notably, the signals increased in the cytoplasm than in the round or elliptical spermatids (C3-C4, arrows). Both in late-stage spermatids and mature sperm, the signal intensity sharply declined, and the sporadic signal was distributed in the opening of the nucleus cup, where the mitochondria were concentrated (D3-D4, E3-E4, arrows). Bar=20 μm.

We used Chinese mitten crab as a model animal. This economic crab belongs to the Arthropoda, Crustacea, Decapoda, Brachyura crab families. The spermatogenesis of Chinese mitten crab is very particular with distinct acrosome, cup-like nucleus and mitochondria ring, which will help to distinguish different stages of spermiogenesis. Therefore, Chinese mitten crab is an ideal model to study the mechanism of mitochondria ubiquitination during spermiogenesis of crustacean. The spermatozoa of *E. sinensis* begin as spermatogonia that subsequently differentiate into spermatocytes and form fine cells through meiosis. During spermiogenesis, the cells undergo a series of morphological changes into sperm. The mature sperm of crustaceans are not flagellated and do not swim. According to morphological changes of the nucleus and acrosome, the spermatogenesis of *E. sinensis* was divided into four stages, including early stage, middle stage, late stage and mature sperm. During the early stages of spermiogenesis, spherical nuclei become half-moon. The pro-acrosome particles are subsequently transported to one pole of the spherical nucleus to form pro-acrosome vesicles (Figure 1, B4). In the middle and late stages, the meniscus nucleus becomes an elongated cupular nucleus, and the gradually increased pro-acrosome develops into the acrosome, acrosome tubule and acrosome complex (Figure 1, C4, D4). There are no obvious organelles in mature sperm, except that the limited number of mitochondria will gradually be arranged in the poles of the cup-shaped nucleus (Figure 1, E4). It's worth noting that the number of mitochondria in the spermatogonia and spermatocytes is more than spermatids. However, in the late stage of spermatids, the mitochondria are degraded and the number is drastically reduced. The remaining limited mitochondria occur directional movement and eventually form a mitochondrial ring invaginated into the nucleus in mature sperm. In this process, how are the mitochondria selected? Mitochondrial degradation mechanisms in Chinese mitten crab is still unknown.

Based on the multiple functions of PHB in mitochondrial biology and its potential role in reproduction, we assume that PHB may regulate spermatogenesis through its mitochondrial membrane protein identity in *E. sinensis*. In recent years, the ubiquitin-proteasome pathway (UPP) and its physiological function in the cell cycle have become research hotspots [29]. Ubiquitination is also widely observed at various stages of spermatogenesis [30]. Importantly, PHB was identified as a substrate for mitochondrial ubiquitination in mice sperm, thereby ensuring maternal inheritance [9, 11]. The polyubiquitin-labeled PHB can accumulate in damaged spermatozoa, suggesting that PHB ubiquitination may mediate mitochondrial degradation [11]. Because of the high degree of conservation of PHB and ubiquitin, we hypothesized that there may be a similar mechanism in crustaceans that will help to clarify the role of PHB in spermiogenesis.

In the present study, we explored one of the physiological mechanisms of spermiogenesis in *E. sinensis*, which was that PHB located on the mitochondrial inner membrane participates in mitochondrial ubiquitination during spermiogenesis of *E. sinensis*. Our results show that PHB and its ubiquitination decide the fate of mitochondria during spermiogenesis of *E. sinensis*.

## RESULTS

### Temporal and spatial expression patterns of *phb* during spermiogenesis in *E. sinensis*

We used FISH to track the distribution of *phb* mRNA during spermiogenesis. The results showed that *phb* mRNA is primarily located in or around the nucleus (Figure 1, arrows). In the round or elliptical spermatocytes, we observed the *phb* mRNA signal in both the nucleus and cytoplasm (Figure 1, A3-A4, arrows). In round or elliptical spermatids, the *phb* mRNA signal was weakly distributed in the nucleus (Figure 1, B3-B4, arrows). In the middle-stage spermatids, the nucleus became cup-like, and the *phb* mRNA signals were distributed in cup-like nucleus and gathered to one side in the cytoplasm. Notably, the signals became stronger in the cytoplasm than in the round or elliptical spermatids (Figure 1, C3-C4, arrows). In both late-stage spermatids and mature sperm, the signal intensity sharply declined, and the sporadic signal was distributed in the opening of the nucleus cup, the area in which mitochondria were primarily concentrated (Figure 1, D3-D4, E3-E4, arrows). The control group was stained by GFP probes without any signal.

### Identification of PHB expression in different tissues of *E. sinensis*

**Figure 2:**
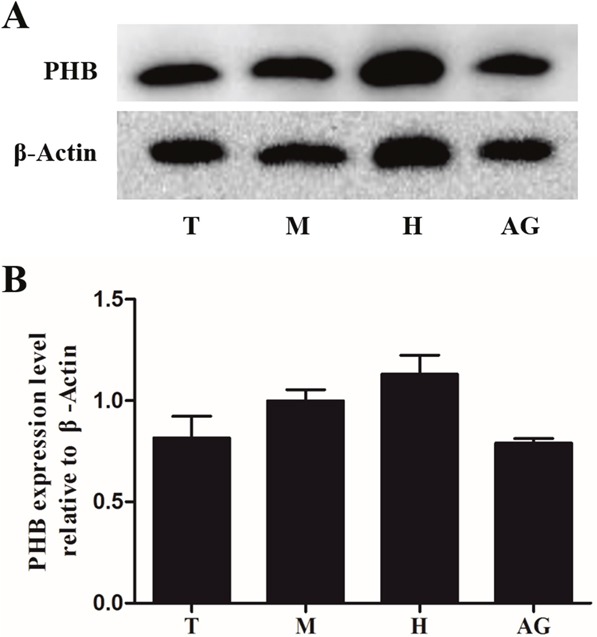
Expression of PHB protein in different tissues of *E. sinensis* The protein was separated using polyacrylamide gel electrophoresis (total protein content: 10μg), and the protein was transferred to nitrocellulose membranes. PHB polyclonal antibody was used to detect the tissue expression. β-actin was used as a control **(A)**. The results showed that PHB was strongly expressed in all the tested tissues **(B)**. T: testis, M: muscle, H: heart, AG: accessory gland.

To detect the titer of anti-PHB polyclonal antibody and the protein expression in different testis samples, western blotting was applied to identify PHB protein expression in the testis, muscle, heart and accessory gland of *E. sinensis*. The anti-PHB polyclonal antibody recognized a single and valid band (36 kDa), which indicated the PHB band (Figure 2, upper panel). The control was treated with an anti-β-actin polyclonal antibody (Figure 2, lower panel). PHB protein was generally expressed in the testis, muscle, heart and accessory gland of *E. sinensis* using quantitative analysis (Figure 2B).

### Mapping of mitochondria and PHB in spermatids

**Figure 3:**
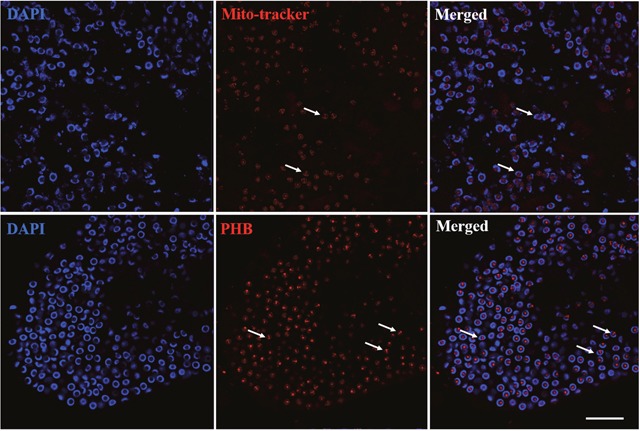
Distribution comparison of mitochondria and PHB during spermiogenesis Mitochondria and PHB were primarily distributed in the acrosome tube and acrosome cap. Bar=10 μm.

To confirm the relationship of mitochondria and PHB during spermiogenesis in *E. sinensis*, we compared their distribution in spermatids. Immunofluorescence analysis revealed that mitochondrial and PHB are primarily distributed in the acrosome tube and acrosome cap, showing highly consistent co-location during spermiogenesis (Figure 3). This finding is consistent with PHB as a mitochondrial membrane protein.

### Expression of PHB and FK2 during spermiogenesis in *E. sinensis*

**Figure 4:**
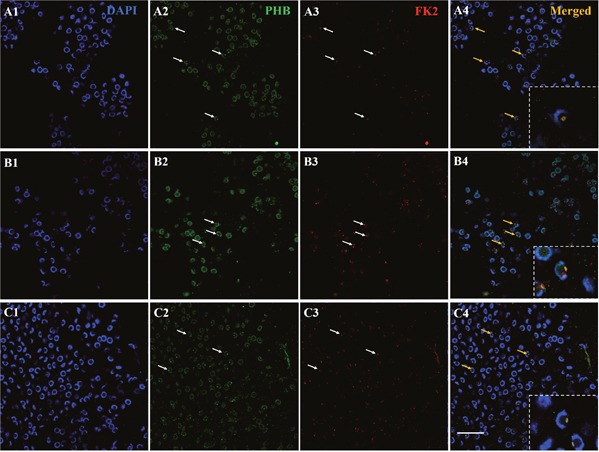
Co-localization of PHB and FK2 expression during spermiogenesis of *E. sinensis* In the early and middle stages, PHB has a few signals surrounding the nucleus and concentrating on one side of the cytoplasm. The FK2 signal is concentrated in the cytoplasm and surrounded by crescent shaped nuclei, consistent with the distribution of PHB in the cytoplasm. The arrows represent early or middle spermatids in A2-A4. In the late stages of spermiogenesis, PHB signals are distributed around the cell membrane and in the acrosome cap. The FK2 signals are primarily concentrated in the acrosome cap. The arrows represent middle and late spermatids in B2-B4. In mature spermatozoa, the nuclei are typically cup-shaped, and the PHB signal is concentrated on the acrosome cap, but the PHB signals in this period are not as intense as in the middle stage. FK2 signals are enriched in acrosome cap and are highly consistent with PHB signal localization. The arrows represent mature sperm in C2-C4. The yellow part of the arrow in the Merged figure shows the co-localization of PHB and FK2. Bar=10 μm.

To determine whether PHB could be the potential substrate of ubiquitination during spermiogenesis, we detected the colocalization of PHB and FK2 using immunofluorescence. In the early and middle stages of spermiogenesis, the nucleus appeared crescent-shaped, as the cytoplasm was gathered on one side of the spermatid. At this stage, PHB has little signal surrounding the nucleus and concentrated on one side of the cytoplasm. The FK2 signal was focused in the cytoplasm and surrounded the crescent-shaped nuclei, consistent with the PHB location in the cytoplasm (Figure 4, A1-A4). In the middle and late stages, the PHB signal covered the acrosome cap and was observed in the cell membrane. During this period, the FK2 signal was primarily concentrated in the acrosome cap, suggesting that polyubiquitin occurs at the acrosome cap (Figure 4, B1-B4). These results suggested that PHB might act as a ubiquitination substrate in ubiquitination. Polyubiquitin may also accumulate at the acrosome cap in preparation for the acrosome reaction. In mature sperm, a typical cup-shaped nucleus was observed, and the PHB signal was primarily concentrated in the acrosome cap. Notably, the PHB signal at this stage was not as strong as that detected in the middle stage (Figure 4, C1-C4), suggesting that the number of mitochondria in mature sperm was significantly reduced. Moreover, the FK2 signal was enriched in the acrosome cap, which was consistent with the PHB signal location.

### The colocalization of mitochondria and FK2 during spermiogenesis in *E. sinensis*

**Figure 5:**
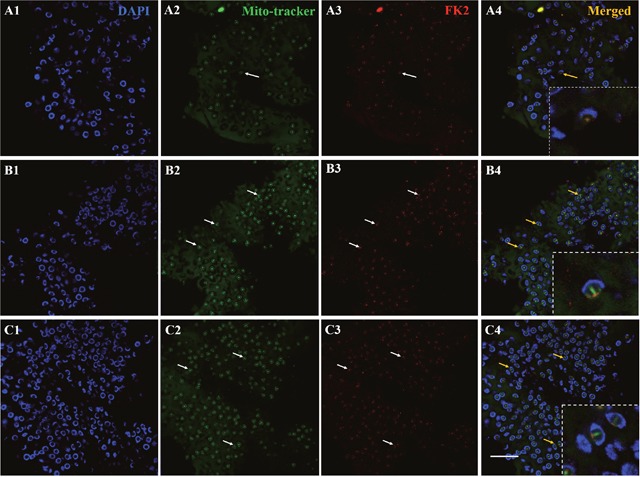
Co-localization of mitochondria and FK2 expression during spermiogenesis of *E. sinensis* In early and middle stages of spermiogenesis, mitochondria are concentrated on one side of the cytoplasm, and FK2 signals are concentrated on the same side of spermatid, consistent with mitochondrial markers in the cytoplasm. In the middle and late stages of spermiogenesis, the signals of mitochondria were concentrated in acrosome cap and acrosome tube, and the signal in acrosome tube was stronger than that in acrosome cap. During this period, the FK2 signals were primarily concentrated in the acrosome cap, and there were also weak signals in the acrosome tube. In mature spermatozoa, the nucleus is typical cup-shaped, and the signals of mitochondria also primarily focus on the acrosome tube and acrosome cap, and the signal in the acrosome tube is stronger. The FK2 signal is also concentrated on the acrosome tube and acrosome cap, but the signal on the acrosome cap is stronger. Mitochondria were partially localized to the FK2 signals. Bar=10 μm.

To study whether redundant mitochondria during spermiogenesis could be degraded through ubiquitination, we detected the colocalization of mitochondria and FK2 using immunofluorescence. In the early and middle stages, the nucleus appears crescent-shaped, and the cytoplasm gathers on one side of the spermatid. The mitochondria were also concentrated on one side of the cytoplasm, and consistently, the FK2 signal was also concentrated on one side of the cell (Figure 5, A1-A4). In the late stage, the mitochondria signal was concentrated in the acrosome tube and acrosome cap. The signal in the acrosome tube was significantly stronger than that in the acrosome cap. During this period, the FK2 signal was primarily concentrated in the acrosome cap, while the acrosome tube had a weak signal, indicating that polyubiquitin is gathered at the acrosome cap (Figure 5, B1-B4). These results suggested that redundant mitochondria might be degraded through ubiquitination. In mature spermatozoa, the mitochondrial signal was primarily concentrated in the acrosome tube and acrosome cap, and the signal in the acrosome was stronger. The FK2 signal was also concentrated in the acrosome tube and the acrosome cap, but the signal in the acrosome cap was stronger. The mitochondria and FK2 signals were partially co-located (Figure 5, C1-C4).

### Effects of PHB knockdown to mitochondrial ubiquitination *in vitro*

**Figure 6:**
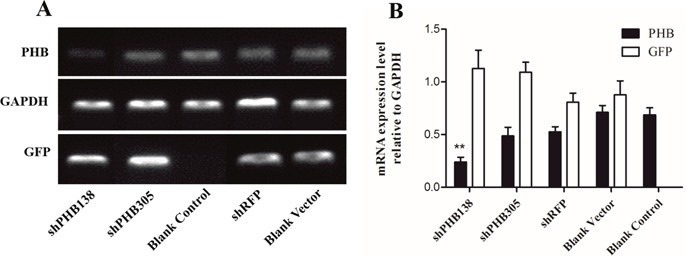
Determination of PHB knockdown in MLTC1 **(A)** The expression of *phb* gene was detected after transient transfection with shRNA interference plasmid for 48 h. GAPDH was used as a positive control. GFP indicates the transfection effect of plasmid. **(B)** Analysis of relative expression of *phb* gene after transient transfection with shRNA interference plasmid for 48 h. The expression of *phb* in the shPHB138 group was approximately 66% of the blank vector group and blank control group. ShPHB305 interference efficiency was approximately 33%. The expression of *phb* in the control groups was significantly higher than that in the shPHB138 group. ^**^P < 0.01.

To detect the relationship between PHB and mitochondrial ubiquitination, we designed *in vitro* experiments using the recombinant plasmids pRNAi-U6.2/Lenti-shPHB138, pRNAi-U6.2/Lenti-shPHB305, pRNAi-U6.2/Lenti-shRFP and the blank vector pRNAi-U6.2/Lenti to transfect MLTC-1 cells for 48 h, respectively. Subsequently, the expression of the *phb* gene was detected to determine the efficiency of interference (Figure 6A). The transfection of the blank vector pRNAi-U6.2/Lenti, nonsense fragment shRFP and blank groups were used as control groups. The gene expression of GFP bands showed that the transfection efficiency in the two interfering groups was significantly higher than that in the control groups, indicating a high-efficiency transfection. The expression of *phb* in the shPHB138 group was approximately 66% of the blank vector and blank control groups, while the efficiency of shPHB305 interference was approximately 33% (Figure 6B). The semi-quantitative results showed that the interference group shPHB138 is more efficient than shPHB305. Therefore, we selected the pRNAi-U6.2/Lenti-shPHB138 interference group for further analysis.

**Figure 7:**
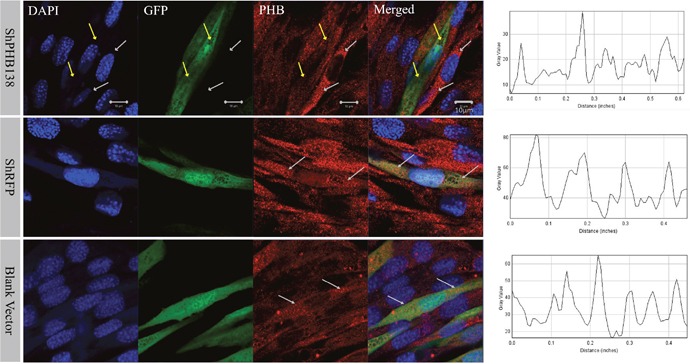
PHB expression and distribution analysis after *phb* knockdown in MLTC-1 cells The expression of PHB in cells treated with shPHB138 was significantly decreased and scattered. The yellow arrows indicate the expression of PHB in transfected cells, and the white arrows refer to the control cells. DAPI was used to label the nucleus, GFP was used to label the transfected cells, and the red signals indicate PHB protein. The last column showed PHB signal strength analysis using ImageJ. Bar=10 μm.

**Figure 8:**
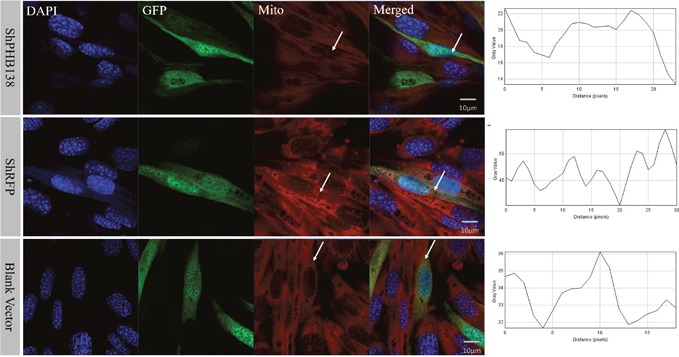
Mitochondrial state and distribution analysis after *phb* knockdown in MLTC-1 cells In the nonsense control (shRFP) and the blank vector control groups, the mitochondria signals were strong and consistent in the transfected and untransfected cells. However, the mitochondria signals in the transfection group were weaker than those in the control groups. The DAPI was used to label the nucleus, GFP indicates the transfected cells, and the red signal is the mitochondrial signal. The last column showed PHB signal strength analysis by ImageJ. Bar=10 μm.

**Figure 9:**
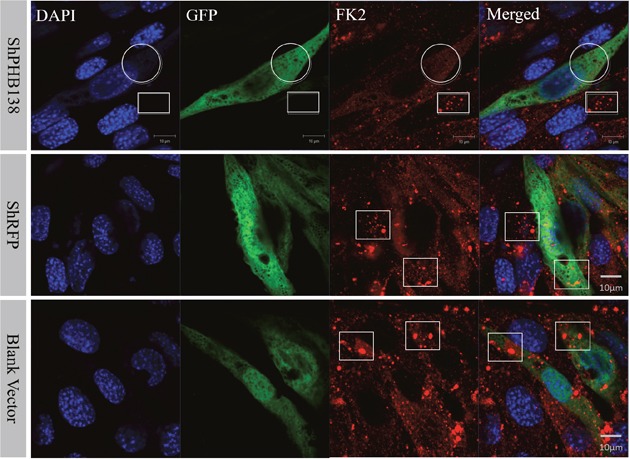
Ubiquitination level analysis after *phb* knockdown in MLTC-1 cells After *phb* gene knockdown, FK2 signal is weak and not group-like aggregation (White Circle). In the interference group, FK2 accumulated in small groups in the untransfected cells (White Box); whereas in the nonsense control (shRFP) and blank vector groups, FK2 was expressed in transfected cells and untransfected cells. DAPI was used to label the nucleus, GFP labeled the transfected cells, and the red signal represents the FK2 signal. Bar=10 μm.

After the transfection of pRNAi-U6.2/Lenti-shPHB138 for 72 h, the expression level of PHB was significantly decreased in the transfected cells (Figure 7, yellow arrows), while the expression level of PHB was not low in the untransfected cells (Figure 7, white arrows). In the nonsense (shRFP) and blank control groups, the expression level of PHB was higher than that in the transfection group (Figure 7, white arrows). The mito-tracker signal was strong and consistent in both transfected and untransfected cells in the control groups after 72 h (Figure 8, white arrows). However, in the transfection group, the mito-tracker signal was weaker than that in the control groups (Figure 8, white arrows). This phenomenon suggested that the reduction of PHB protein significantly affected the mitochondrial state. In the transfected cells, the FK2 signal was weaker (Figure 9, Circles), whereas FK2 accumulated in small amounts in untransfected cells or the control groups (Figure 9, Boxes). This phenomenon suggests that the reduction in PHB has a significant effect on polyubiquitination levels.

### Effects of *es-phb* overexpression to mitochondrial ubiquitination *in vitro*

**Figure 10:**
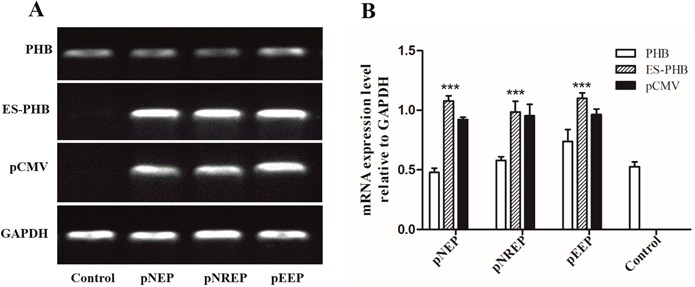
Determination of *es-phb* overexpression in MLTC1 cells **(A)** The expression of *phb* and *es-phb* gene after transient transfection for 48 h. GAPDH is the positive control. pCMV was used to determine the effect of plasmid transfection. **(B)** Analysis of relative expression of *es-phb* gene after transient transfection for 48 h. The expression level of *phb* gene indicates the intracellular expression level of intracellular level. *es-phb* gene indicates overexpression gene expression. pCMV was detected as an indicator of transfection efficiency. pNEP: pCMV-N-Flag-es-phb, pNREP: pCMV-N-Flag-RFP-es-phb, pEEP: pCMV-EGFP-es-phb. ^***^P < 0.001.

**Figure 11:**
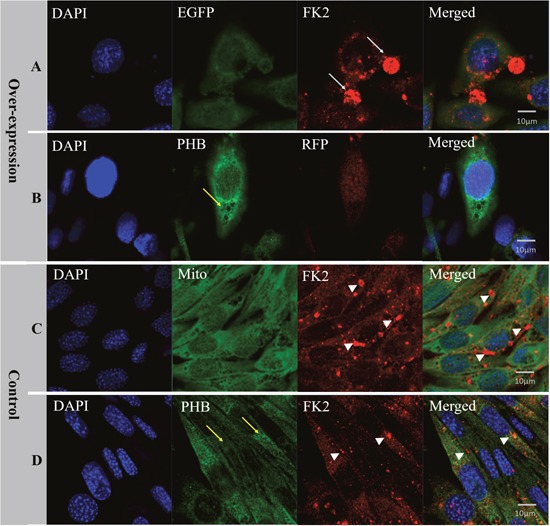
Analysis of PHB expression, mitochondria state and ubiquitination level after *es-phb* overexpression in MLTC-1 cells **(A)** The group transfected with the expression plasmid pCMV-N-Flag-RFP-es-phb. **(B)** The group transfected with the expression plasmid pCMV-EGFP-es-phb; **(C)** and **(D)** The control group. The expression level of PHB in overexpression group was higher than that in the control group (yellow arrows). In the overexpression group, FK2 was clustered into large clumps (white arrows), while FK2 in the control group was clustered into small agglomerates (white arrow heads), indicating that PHB overexpression increased the poly-ubiquitin level. Bar=10 μm.

To verify the function of PHB in mitochondrial ubiquitination, we overexpressed *es-phb* (*phb* in *E. sinensis*) into MLTC-1 cells. The three recombinant overexpression vectors pCMV-N-Flag-es-phb, pCMV-N-Flag-RFP-es-phb, and pCMV-EGFP-es-phb were transfected into MLTC-1 cells for 48 h. The expression of the *phb* and *es-phb* genes was examined (Figure 10A). GAPDH was used as a positive control. The pCMV segment was detected as an indicator of transfection efficiency. The expression levels of pCMV and *es-phb* in the three overexpression groups were higher than those in the control group, indicating that the overexpression plasmid could be successfully transferred into the cells, and the transfection efficiency was high (Figure 10B). The *es-phb* band was not detected in the control group, indicating that the *es-phb* primer was specific, and *es-phb* was overexpressed (Figure 10A). After transfection for 72 h, the expression level of PHB protein was higher than that in the control group. In the overexpression group, FK2 was clustered in large clumps, while FK2 was clustered in small agglomerates in the control group (Figure 11, white arrows), indicating that the overexpression of PHB protein increased the level of mitochondrial polyubiquitination.

## DISCUSSION

The formation of acrosomes and the deformation of the nucleus are two key events in spermiogenesis in crustaceans. The cup-shaped nuclei and nuclei extending radiator arms are the salient features of the sperm of such species. The distribution of *phb* mRNA in the spermatozoa of *E. sinensis* showed a signal located in the small gap between the nucleus and acrosome, consistent with the results of the FISH analysis in late stage spermiogenesis. These results indicated that *phb* might play a role in acrosome formation and nuclear deformation. However, PHB protein function has not been further studied. In the present study, we investigated the role of PHB in mitochondrial ubiquitination using *in vivo* and *in vitro* methods, respectively. *in vivo* experiments using Western blotting and immunofluorescence showed that PHB was involved in mitochondrial ubiquitination degradation. *In vitro* experiments, the effects of PHB on mitochondrial ubiquitination were further investigated using shRNA interference and *es-phb* protein overexpression in MLTC-1 cells.

### Role of PHB in mitochondria deformation, acrosome formation and nuclear shaping in crustaceans

Western blotting showed that the expression of PHB protein in the tested tissue was relatively high, consistent with PHB as a mitochondrial membrane protein or chaperone [31, 32]. The relative expression of PHB in the accessory gland was lower than that in the testis, reflecting the storage of mature sperm in the accessory glands and the storage of spermatids in the testis at different stages. Consistent with *P. clarkii*, the expression of PHB in sperm was higher than that in mature sperm [26]. In early and middle sperm cells, acrosome formation and nucleus deformation require mitochondria to provide large amounts of energy; thus, the abundant expression of PHB could ensure spermatid development. In mature sperm, the cell composition is fairly low. Only a small number of mitochondria are distributed at both ends of the nucleus (Figure 5), and PHB expression is also reduced.

PHB was detected in the cytoplasmic membrane, nuclei and mitochondria [16]. In *Homo sapiens*, *R. norvegicus*, *S. cerevisiae* and other species, PHB was identified as a mitochondrial inner membrane protein [23–28]. PHB plays intracellular roles through the formation of similar ring-like PHB complexes embedded in the mitochondrial membrane [33, 34]. In the present study, we examined the function of mitochondrial inner membrane protein PHB during spermiogenesis in *E. sinensis*. The distribution analysis during spermiogenesis showed that the co-localization of PHB and mitochondria was highly consistent (Figure 3). PHB, located on mitochondria based on mitochondrial m-AAA proteases, was involved in the regulation of membrane protein degradation. PHB may also play a role in unfolding enzyme partners to facilitate non-covalent protein folding, maintaining the stability of unassembled membrane proteins [31, 32, 35]. In addition, PHB regulates cell differentiation and apoptosis through the recruitment of lipid-associated membrane proteins, which plays an important role in the construction of mitochondrial ridge [17, 33, 36]. During spermiogenesis in *E. sinensis*, mitochondria undergo a decrease in mitochondria number and show changes in the structure and transportation of these organelles. Immunofluorescence analysis showed that PHB was highly expressed in spermatids at different stages, indicating that PHB participated in the growth of mitochondrial crests (Figure 4). The high co-localization of PHB and mitochondria reflects the indispensable role of PHB plays in stabilizing the mitochondrial genome [37–39]. Taken together, the FISH and immunofluorescence analyses the present study showed that during the early and middle stages of spermiogenesis, *phb* gene and PHB protein are primarily focused in pro-acrosomes and demilune nuclei, showing higher expression (Figure 1, Figure 4). Studies have suggested that PHB may play a role in acrosome formation and nucleus shaping. The potential mechanism for this phenomenon is that PHB promotes mitochondria deformation and maintains mitochondrial function during spermiogenesis to ensure that mitochondria provide sufficient energy.

### PHB mediates mitochondrial ubiquitination during spermiogenesis

The latest studies have shown that mitochondria are targets of polyubiquitin [40, 41]. Studies have also shown that ubiquitinated PHB is present in mammalian sperm mitochondria, suggesting that this protein may mediate the ubiquitination of spermatozoa mitochondria [11]. However, it remains unknown whether this physiological mechanism mediates spermatogenesis in crustaceans.

In the early and middle stages of spermiogenesis in *E. sinensis*, the nucleus appears crescent-shaped, and the cytoplasm is clustered on one side of the spermatid (Figure 1 B4 and C4). At this period, *phb* surrounds the nucleus and is concentrated on one side of the cytoplasm. FK2 has a low signal focused on the formation of the acrosome side, surrounded by crescent-shaped nuclei, consistent with the distribution of PHB and mitochondria (Figure 4, A2; Figure 5, A2). Additionally, the ubiquitination level of PHB and mitochondria is relatively low (Figure 4, A3; Figure 5, A3). In the late stage of spermatogenesis, PHB and mitochondrial markers cover the acrosome and nucleus (Figure 4, B2; Figure 5, B2). The FK2 signal is gradually increased and primarily distributed in the acrosome cap and on one side of the nucleus (Figure 4, B3; Figure 5, B3), suggesting that the polyubiquitination level increases in the late stage. There are also a small number of mitochondria in the acrosome, suggesting that some mitochondria are specialized as part of the acrosome. PHB, mitochondria and FK2 show co-localization at the acrosome cap and on one side of the nucleus, indicating that mitochondria are polyubiquitinated at a high level. The mature sperm have typical cup-shaped nuclei (Figure 1, E4). The mitochondria are distributed in acrosome tubes and show co-localization with PHB (Figure 5, C2). The PHB signal is concentrated in the acrosome cap and cup-shaped nucleus (Figure 4, C2). However, during this period, the PHB signal is not as strong as in the middle stage, likely reflecting a significant reduction in the number of mitochondria in mature spermatozoa. The FK2 signal is enriched in the cap body, consistent with PHB and mitochondrial positioning (Figure 4, C3; Figure 5, C3). The polyubiquitin may also accumulate in the acrosome cap in preparation for the acrosome reaction.

According to the present study, during spermiogenesis, the co-localization of PHB with mitochondrial and polyubiquitin markers (FK2) suggests that PHB may be used as a substrate in ubiquitinated mitochondrial degradation, or the polyubiquitin chain specifically labels mitochondria through PHB. Thus, PHB-mediated mitochondrial ubiquitination may be a conserved mechanism during spermiogenesis.

Studies have shown that knockdown or knockout of PHB in yeast or mammalian cells results in the cleavage of highly coherent tubules in mitochondria, reflecting membrane fusion and a splitting imbalance [25, 42]. These results also suggest that PHB is a necessary factor for the fusion of mitochondrial membranes [36]. The knockout of PHB results in sterile gamete-deficient individuals [25]. In addition, studies have shown that PHB transcription is initially detected in rat spermatogonia, and PHB proteins are expressed throughout germ cell development [23]. In mammalian spermatozoa, PHB is considered a substrate for spermatozoa mitochondrial polyubiquitination [11].

To study the effects of PHB knockdown on mitochondria and ubiquitination, we used *in vitro* interference experiments. *In vitro phb* interference experiments showed that FK2 was not be clustered (Figure 9), and the mitochondrial signal was also reduced (Figure 8); and *es-phb* overexpression experiments showed that FK2 was clustered in large chunks, and only a small range of signals were clustered in the control cells (Figure 11). *In vitro* experiments showed that PHB has an important effect on the level of ubiquitination in cells. The PHB overexpression experiment showed that as a mitochondrial inner membrane protein, the expression of PHB significantly reduced mitochondrial functions and stability, and defective mitochondria undergo lysosomal degradation or ubiquitination degradation. However, in the interference group, the level of ubiquitination was significantly reduced, likely reflecting the significantly decreased expression of the polyubiquitin substrate PHB. This phenomenon fully demonstrates that the mitochondrial membrane protein PHB acts as a polyubiquitin substrate involved in mitochondrial ubiquitination.

### Mitochondrial ubiquitination in spermiogenesis of mammal and Chinese mitten crab

During spermiogenesis, mitochondrial ubiquitination has important biological significance. It has been reported that ubiquitinated mitochondrial proteins are protected within mitochondrial sacs with a disulfide cross-link prior to fertilization in mammal [10]. After fertilization, the disulfide bonds are reduced in the environment of the fertilized eggs, thus these proteins are exposed and degraded [10]. Subsequently, the mitochondria of the male parent are degraded through the ubiquitin-proteasome pathway in the fertilized egg; otherwise, the remaining parental mitochondria will interfere with mitochondrial maternal inheritance [43–45]. The results of the present study are consistent with previous studies in mammals. In the present study, PHB, mitochondria and polyubiquitin chains showed significant co-localization. The signals were reduced in the early stage, peaked in the middle stage and remained high in the late stage, suggesting that the mitochondrial membrane protein PHB may be gradually labeled with polyubiquitin from the late stage. These polyubiquitin chains are prepared for mitochondrial degradation after fertilization, thereby ensuring paternal mitochondria disintegration and maternal inheritance in Chinese mitten crabs.

In the present study, we present a physiological mechanism during spermiogenesis in *E. sinensis*. Firstly, PHB plays an important role in maintaining the stability of mitochondria. Secondly, PHB functions as a mitochondrial membrane protein during acrosome formation and nucleus shaping. Thirdly, PHB acts as a ubiquitination substrate to mediate mitochondrial ubiquitination in spermatids and may have a more important function in spermiogenesis. Simultaneously, the present study provided an ideal model for the molecular mechanism of organelle ubiquitination during spermiogenesis in crustaceans.

## MATERIALS AND METHODS

### Fluorescence *in situ* hybridization (FISH)

**Tissue preparation.** The testis of *E. sinensis* were dissected and embedded in O.C.T (optimum cutting temperature) compound for storage at −40°C and sectioning at 6-μm thickness using a sliding microtome. The sections were adhered onto RNase-free poly-L-lysine-coated slides, and immediately stored at −80°C until subsequent use in FISH analyses.

**Riboprobe synthesis.** Specific mRNA probes were designed from the open reading frame of the *phb* gene using Primer 5.0, and these probes were synthesized at Generay Biotechnology (Shanghai, China). The sequences were conjugated with green fluorophores: 5’-FITC-TAGGCTTCGGTGTCGCTGTCGT for phb and 5’-FITC-GAGTTCAAGTCCATCTACATGG for GFP as control.

**Hybridization.** The testis sections were dried at room temperature for 10 min and fixed with 4% paraformaldehyde (PFA in PBS, pH 7.4) for 10 min. Subsequently, the sections were rinsed twice for 10 min in 0.1% DEPC-activated 0.1 M PBS (pH 7.4) at room temperature, and permeabilized with 0.2% PBST (0.1 M PBS in Triton) for 30 min. The *phb* and GFP probes were diluted to 1 μM in PBS and incubated with the sections for 2 h at room temperature in the dark. Subsequently, the sections were washed 3 times with PBS for 45 min. Subsequently, all sections were stained with DAPI (Beyotime, China) for 5 min to indicate the nucleus. Finally, the sections were mounted in Antifade Mounting Medium (Beyotime, China) and observed using a confocal laser-scanning microscope (CLSM 510) (Carl Zeiss, Germany).

### Preparation of recombinant proteins

Sequences encoding phb were amplified using PCR with two specific primers (Table 1) containing a BamHI site and an EcoRI site added on the 5’ and 3’ ends, respectively. The resulting amplifications of phb were ligated to pET28a (Invitrogen Life Technologies, USA). Plasmid DNAs were transformed into DE3 (Novagen, Germany). A single colony of DE3 harboring the targeting plasmids was inoculated into 10 ml of Luria-Bertani (LB) medium supplemented with kanamycin (Kana^+^) (25 mg/l) and shaken at 37°C for 6-8 h. The pre-culture broth was added to LB medium (Kana^+^) at a ratio of 1: 100, and shaken at 200 rpm at 37°C for several h until the OD600 value reached 0.4-0.8. Isopropyl-β-d-thiogalactoside (IPTG) was added at a final concentration of 1 mM, and the culture was continually incubated at 37°C and shaken at 200 rpm for 10-12 h. After ultrasonication and centrifugation, the sediment was collected and re-suspended with PBS. Recombinant His-PHB proteins in these re-suspended liquids were purified using Ni-Agarose His according to the manufacturer's instructions (CWBIO, China) and assessed using 10% SDS-PAGE.

**Table 1:** The list of all primers used in the study

Primer name	Sequence	Purpose
BDPHBF1	CGCGGATCCCTCAACTCCGCCCTCTACAAT	Prokaryotic expression
BDPHBR1	CCGGAATTCTCCTCTGATGCCTCAATCCT	Prokaryotic expression
T7F	TAATACGACTCACTATAGGG	Prokaryotic expression
T7R	TGCTAGTTATTGCTCAGCGG	Prokaryotic expression
pCMV-F	AATTAACCCTCACTAAAGGG	Vector primers
pCMV-R	GTAATACGACTCACTATAGGGC	Vector primers
PHB-F	CAGGAGGCGTGGTGAACT	Semi-quantitative (SQ)
PHB-R	CGGAAGAGGATTCGCAGT	SQ
GAPDH-F	ACCACAGTCCATGCCATCAC	SQ
GAPDH -R	TCCACCACCCTGTTGCTGTA	SQ
ES-PHB-F	CGCGGATCCATGGCGCAGCAGCTGGCCA	Overexpression and SQ
ES-PHB-R	CCGGAATTCGAGGCAAGGACAGGAGGGTAC	Overexpression and SQ
pU6.2-F	CAGTGCAGGGGAAAGAATAGTAGAC	Semi-quantitative
pU6.2-R	TCGACCTGCTGGAATCTCGTG	Semi-quantitative

### Preparation of Ab

The purified Recombinant His-PHB proteins was sent to Hangzhou HuaAn Biotechnology Co., Ltd. to prepare a rabbit polyclonal Ab. Western blot analyses were performed to characterize the specificity of the Ab, in which 10μg of testis extract was used.

### Antibodies

The PHB polyclonal antibody was prepared at Hangzhou HuaAn Biotechnology Co., Ltd. (China). Rabbit GAPDH and anti-β-actin was purchased from BIOS (Shanghai, China). The Mito-Tracker Green/Red was purchased from Invitrogen (California, USA). FK2 was purchased from Stressgen (Canada). HRP conjugated goat anti-rabbit IgG and Alexa Fluor 488-labeled goat anti-rabbit IgG were purchased from Beyotime Biotechnology (Shanghai, China). Texas Red-labeled goat anti-mouse and goat anti-rabbit IgG were purchased from Jackson Immuno Res Lab (USA).

### Western blot analysis

Different tissue extracts with equal amounts of protein were separated using 10% gels (SDS-PAGE) and transferred onto the PVDF membranes (Bio-Rad, California, USA). After blocking with 5% non-fat milk in 0.5% PBST (PBS with 0.5% Tween 20) for 1 h, the membranes were incubated overnight at 4°C with rabbit anti-PHB antibody (diluted 1:500 with 5% non-fat milk). Anti-β-Actin antibody (1:1000) was used as a loading control. Subsequently, the membranes were incubated with secondary HRP-conjugated antibody (diluted 1:1000) and visualized using chemiluminescence imaging (Chemiscope 3400) (Shanghai, China).

### Cell culture and treatment

MLTC-1 (Mouse testicular Leydig tumor cells) were obtained from ATCC (ATCC No. CRL-2065) and cultured in 90% RPMI-1640 (GIBCO), supplemented with 10% FBS (GIBCO) and 100 U/ml penicillin-streptomycin (GIBCO) at 37°C with 5% CO_2_.

### RNAi knockdown and transient transfection

The sequences for shRNA targeting *phb* were *phb* shRNA-138 (shPHB138), 5’-GCTTCCTCGTATCTACACC-3’; *phb* shRNA-305 (shPHB305), 5’-GGACATTGTGGTCGGGGAA-3’; the sequence of control Luciferase shRNA (shRFP) was 5’-GAGTTCAAGTCCATCTACA-3’. The synthesized shRNAs were subjected to oligonucleotide annealing and phosphorylation and finally constructed into pRNAi-U6.2/Lenti vector plasmids (Biomics, China). According to the manufacturer's instructions (Life Technologies), for a 6-well plate, 8 μl of Lipofectamine 2000 reagent was diluted in 240 μl opi-MDM reduced serum medium for 5 min at 37°C. Subsequently, 2.5 μg recombinant plasmid was diluted in 240 μl opi-MDM reduced serum medium (GIBCO). The two solutions were mixed and incubated for 20 min at 25°C. Subsequently, the above transfection components were added dropwise into the culture medium in a 6-well plate. After transfection, the cells were collected for subsequent analysis at 48 or 72 h.

### Overexpression of *es-phb* gene in MLTC-1 cells

In the overexpression study, pCMV-N-Flag was purchased from Beyotime (Shanghai, China). pCMV-EGFP and pCMV-N-Flag-RFP were kind gifts from Dr. Zhen-Yu She. The full-length *phb* with restriction enzyme cutting sites (BamHI and EcoRI) was cloned from the testis of *E. sinensis*. The *es-phb* gene was respectively constructed into pCMV-N-Flag, pCMV-N-flag-RFP and pCMV-EGFP. The protocol for transient transfection was conducted in the same manner as used for RNAi knockdown. After transfection, the cells were collected for subsequent analysis at 48 or 72 h.

### Immunofluorescence and confocal microscopy

**Tissues**: The testis of adult male *E. sinensis* was fixed in 4% paraformaldehyde (PFA, pH 7.4) overnight. After rinsing three times in PBS for 15 min, the samples were incubated overnight in 0.5 M sucrose dissolved in PBS and embedded into an O.C.T compound for storage at −40°C, followed by sectioning at 6-μm thickness using a sliding microtome. **Cells**: The cells were fixed in 4% paraformaldehyde (PFA, pH 7.4) for 10 min at room temperature and were subsequently washed three times in PBS for 15 min. The tissue sections or cells were permeabilized with 0.3% PBST (Triton X-100 in PBS) for 10 min at room temperature and subsequently blocked with 5% BSA in PBST (0.1% Triton X-100) for 1 h. The tissue sections or cells were incubated with anti-PHB antibodies (1:100 dilution) or Anti-Poly Ubiquitin mAb (FK2) (1:100 dilution) in 5% BSA at 4°C overnight. After rinsing 3 times in PBST for 45 min, the tissue sections or cells were subsequently incubated with Texas Red goat anti-rabbit IgG (1:100 dilution) or FITC-anti-mouse IgG (1:100 dilution) or Mito-tracker Green/Red (1:1000) for 1 hour at room temperature. After washing 3 times in PBST (0.1% Triton X-100) for 45 min, the nuclei were stained with DAPI (Beyotime, China) for 5 min. Finally, the sections or cells were mounted in Antifade Mounting Medium (Beyotime, China) and observed with a confocal laser-scanning microscope (CLSM 510) (Carl Zeiss, Germany).

## Abbreviations

PHB: prohibitin
TFAM: Transcription Factor A, Mitochondrial
ROS: Reactive Oxygen Free Radical
UPP: ubiquitin-proteasome pathway
FISH: fluorescence *in situ* hybridization
IPTG: isopropyl-β-d-thiogalactoside
MLTC-1: Mouse testicular Leydig tumor cells
EGFP: enhanced green fluorescent protein
RFP: red fluorescent protein
h: hour
